# The effect of Baduanjin on the balancing ability of older adults: A systematic review and meta-analysis

**DOI:** 10.3389/fmed.2022.995577

**Published:** 2022-10-28

**Authors:** Linxuan Guo, Zhihao Liu, Wenxue Yuan

**Affiliations:** School of Kinesiology and Health Promotion, Dalian University of Technology, Dalian, China

**Keywords:** older adults, Baduanjin, balancing ability, meta-analysis, randomized control trial

## Abstract

**Purpose:**

To systematically evaluate the effect of Baduanjin on the balancing ability of older adults.

**Methods:**

The systematic review and meta-analysis followed the Preferred Reporting Items for Systematic Reviews and Meta-Analysis (PRISMA) guidelines. Six electronic databases were searched for eligible studies. Data synthesis and statistical analysis using a random effects model were performed with Review Manager 5.4. Random-effects weights were used to pool the effect sizes. Publication bias was assessed by funnel plot.

**Results:**

A total of 17 RCTs involving 1,267 patients were identified. The meta-analysis showed that the Baduanjin group was significantly superior to the control group in balancing performance measured by Berg balance scale [mean difference (MD) 4.82; 95% confidence interval (CI) 3.40 to 6.24, *P* < 0.00001], Timed Up and Go (MD −2.21, 95% CI −2.69 to −1.74, *P* < 0.00001) and Eye Closed One Leg Standing Balance (MD 2.01, 95% CI 0.79 to 3.23, *P* < 0.00001) tests.

**Conclusion:**

Baduanjin can effectively affect the balancing ability of older adults. More high-quality evidence-based studies are required to confirm these findings.

**Systematic review registration:**

[https://www.crd.york.ac.uk/prospero/display_record.php?RecordID=293183], identifier [CRD42021293183].

## Introduction

The ability to balance generally declines with age because of reduced muscle strength, bone loss, and cognitive decline. Thus, older adults have a high risk for falling than other age groups. Falls can cause soft tissue injury, bone fractures and psychological trauma, leading to disability or even death (1, 2). Falls due to the decline in balance can impact older persons’ life expectancy as well as their quality of life (QOL) and place a greater burden on their family and society in terms of medical costs (3). According to the seventh National Census in China in 2020, 13.5% of the population was aged ≥ 65 years (4), a higher percentage than in previous censuses, indicating that population of China is aging. Methods to enhance the balancing ability of older adults and prevent accidental falls are regard to effectively affect their wellbeing and QOL.

Qigong exercise has been an essential component of traditional Chinese medical care for more than 2000 years (5). Baduanjin, a light-to-moderate intensity Qigong practice, is distinguished by its therapeutic effects for health promotion (6). It emphasizes calm, deep breathing, physical stretches, and mental attention while emphasizing the mind-body connection (7). Baduanjin has been shown to increase the limb strength of middle-aged and older adults and to improve their joint flexibility and balancing ability (5, 6). Baduanjin has also been used to treat patients with osteoporosis and Parkinson’s disease, effectively improving their balancing and limb movement abilities (7, 8).

Although there is some systematic analysis suggesting that Baduanjin can improve the balancing ability of older persons (9), Assessment of Multiple Systematic Reviews 2.0 (AMSTAR 2.0) found that the research was of low methodological quality and evaluated to have highly heterogeneous populations. Moreover, the previous research did not include relevant studies from outside China. The present study was designed to comply with the requirements of AMSTAR 2.0 (10), using strict inclusion and exclusion criteria, to provide more rigorous evidence to systematically analyze the effect of Baduanjin exercises on the balancing ability of older adults.

## Methods

The study protocol for this systematic review and meta-analysis was registered on PROSPERO (International Prospective Register of Systematic Review) with the register number (CRD42021293183) and complied with the PRISMA guidelines.

### Eligibility criteria

The Population, Intervention, Comparison, Outcome and Study (PICOS) framework was used to determine the inclusion criteria for studies and the following selection criteria were applied: (P) Participants: the elderly aged ≥ 60 years, with no restrictions on gender, race, nationality, or living environment; (I) Intervention: studies with Baduanjin as the main exercise intervention in the experimental group, including intervention process, duration, frequency, and the training length ≥ 4 weeks; (C) Comparator: the control group who have no exercise habit or performed some simple daily activity; (O) Outcome: the scores of Berg Balance Scale (BBS), Timed Up and Go (TUG), and Eye Closed One Leg Standing Balance (ECLSB) tests; (S) Study design: randomized controlled trials (RCT). Studies were excluded if (1) not written in Chinese or English; (2) full texts unavailable; (3) repeated publications; (4) missing original research data or no way to obtain them; or (5) the drop-out rate over 20%.

### Information sources and search strategy

Electronic databases, including PubMed, Web of Science, The Cochrane Library, China National Knowledge Infrastructure (CNKI), WanFang Data, and SinoMed, were searched through November 2021 to identify RCTs assessing the effect of Baduanjin on the balancing ability of older adults. Keywords used for searching included (“Baduanjin” OR “Ba Duan Jin” OR “Ba-Duan-Jin” OR “eight section brocades”) AND (“postural balance” OR “musculoskeletal equilibrium” OR “accidental falls” OR “fall”) AND (“elderly” OR “old man”) AND (“RCT” OR “controlled trial” OR “randomized clinical trial”). The search strategy is illustrated in Supplementary material.

### Data collection process

Studies identified by the online search were independently screened by two researchers, based on the pre-determined inclusion and exclusion criteria, with discrepancies resolved by consensus or consulting a third researcher. Data extracted from these studies included the research objective, sample size, intervention measures, control measures, intervention frequency, intervention duration, outcome indicators, and evaluation of bias risk.

### Statistical analysis

Data synthesis and statistical analysis using a random effects model were performed with Review Manager (version 5.4) available from Cochrane. The mean difference (MD) was adopted as the effect index, with each effect quantity including its point estimate and 95% confidence interval (CI). The statistical heterogeneity among research results was evaluated by χ^2^ tests, with a test level of α = 0.1, which is combined with *I*^2^ to quantitatively assess the extent of heterogeneity (*I^2^* < 25% means low heterogeneity; 25% < *I^2^* < 50% means medile heterogeneity; *I^2^* > 50% means high heterogeneity). Sensitivity analysis was performed to detect the dependency of the overall heterogeneity on a particular study. To identify the probable sources of heterogeneity, subgroup analyses were conducted according to the health status of the participants (healthy elderly vs. frail/transitional elderly) of the included studies. Also, to explore whether total exercise amount impacts the effect of Baduanjin practice, the stratified subgroup analysis was carried out to explore the dose-response relationship for the included studies. Given that the majority of studies did not discuss the intensity of Baduanjin exercises and that all Baduanjin practice followed the same movement paradigms, the study assumed that the intensity of Baduanjin workouts in every study was, by default, consistent. On the basis of total exercise amount, the included studies were classified into three subgroups: small exercise group (SE = exercise time less than 1999 min), medium exercise group (ME = exercise time between 2000 and 3999 min), and large exercise group (LE = exercise amount of 4000–5999 min). The total exercise amount was calculated by duration of weeks, sessions per week and time per session (take the median if it is a range) as listed in Table 1.

**TABLE 1:** Characteristics of the included studies.

Author	Participant description				Intervention protocols		Control group/Condition	Outcomes
		
	Sample size	Age (years) (Mean ± SD)	Female (BDJ/Con)	Source of participants	BDJ	Duration, sessions with supervision per week, time per session		
Shi et al. (11)	64/65	67.89 ± 4.63/67.48 ± 4.52	31/30	Outpatient	BDJ and balance Ex	8 weeks, 4, 64 min	Balance Ex	①
Zhang et al. (12)	38/40	67.84 ± 4.94/68.13 ± 4.67	38/37	Community	BDJ and resistance Ex	12 weeks, 5, 60–90 min	Educational program	②
Zhao (13)	17/17	65.82 ± 3.88/64.35 ± 3.62	–	Community	BDJ	12 weeks, 3, 60 min	No treatment	③
Li et al. (14)	36/35	65.2 ± 3.6/65.7 ± 3.7	–	Community	BDJ	24 weeks, 5, 60 min	Educational program	①
Zhang et al. (15)	42/41	66.68 ± 2.53/66.59 ± 2.73	16/15	Outpatient	BDJ and balance Ex	12 weeks, 7, 34 min	Balance Ex	③
Zhou et al. (16)	20/20	72.67 ± 9.56/73.25 ± 8.54	12/11	Community	BDJ	8 weeks, 5, 40 min	Educational program	① ②
Gao et al. (17)	34/34	79.79 ± 4.18/78.88 ± 4.66	26/28	Nursing home	BDJ	12 weeks, 5, 30 min	No treatment	②
Song et al. (18)	60/60	67.5 ± 3.5/68.2 ± 3.3	28/24	Outpatient	BDJ and balance Ex	8 weeks, 5, 35 min	Balance Ex	①
Wang et al. (19)	42/42	66.40 ± 4.90/66.60 ± 4.70	20/18	Inpatients	BDJ	12 weeks, 5, 30 min	Walking	①
Kuang (20)	41/41	68.68 ± 3.22/70.33 ± 3.34	–	Outpatient	BDJ	12 weeks, 7, 120 min	Medical treatment	①
Li et al. (21)	44/44	65.1 ± 5.1/65.1 ± 5.1	31/29	Outpatient	BDJ	24 weeks, 7, 30–40 min	No treatment	① ② ③
Wu et al. (22)	60/60	70.63 ± 4.52/70.55 ± 4.26	42/42	Community	BDJ	4 weeks, 7, 120 min	No treatment	① ②
Chen et al. (23)	20/20	64.10 ± 2.64/63.00 ± 3.00	8/12	Community	BDJ	12 weeks, 6, 30 min	No treatment	③
Hou (24)	20/20	60–69	–	Community	BDJ	12 weeks, 5, 60–70 min	No treatment	② ③
Liu (25)	7/8	82.14 ± 1.68/84.15 ± 2.95	–	Outpatient	BDJ	12 weeks, 5, 60 min	No treatment	②
He et al. (26)	40/40	63.4 ± 1.5/62.2 ± 2.1	40/40	Women veterans	BDJ	20 weeks, 7, 45 min	No treatment	③
Liu et al. (27)	47/48	≥ 60	38/37	Community	BDJ	12 weeks, 7, 30–40 min	Walking	② ③

BDJ, Ba Duan Jin; Con, control condition/group; Ex, exercise; ①, BBS; ②, TUG; ③, ECLSB.

## Results

### Search selection

**FIGURE 1:**
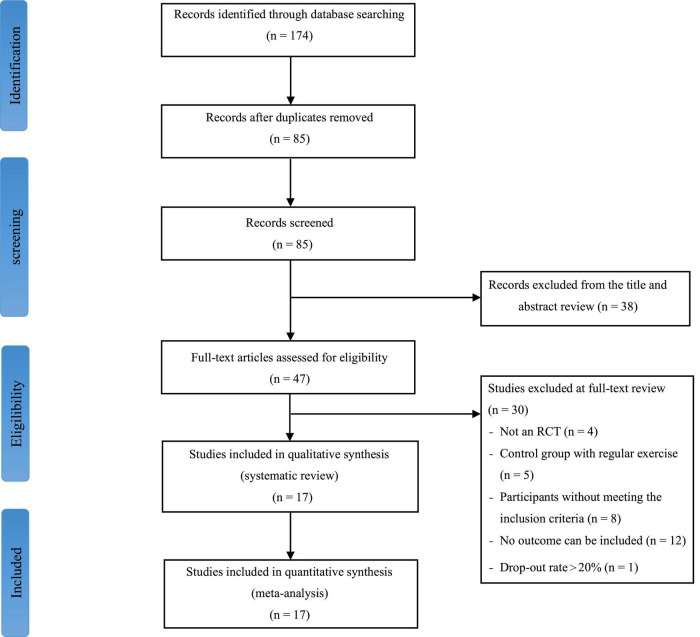
Flowchart of the process of screening and study selection.

One hundred seventy-four studies were initially identified from the electronic databases. Of these, 89 were duplicate studies and excluded. A total of 85 studies were screened for titles and abstracts, and 38 were excluded. After the first stage of screening, 47 studies were selected for full-text screening. Of these, 30 articles that failed to meet the PICOS framework were excluded. As a result, 17 studies were included in the meta-analysis. The flowchart of the process of screening and study selection was illustrated in Figure 1.

### Quality assessment

**FIGURE 2:**
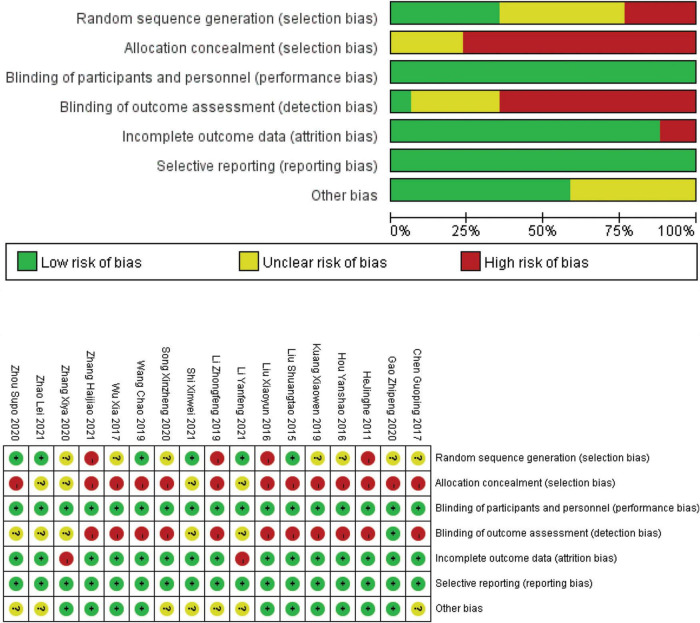
Risk of bias graph and summary.

The quality of the included studies was assessed using the Cochrane Handbook for Systematic Reviews of Interventions. The included studies that recorded inadequate information about the methods used for allocation concealment were rated as unclear or high risk. The subjects and personnel were not blinded due to the particularity of the intervention approaches, which made the relevant trials result in high risk in the performance bias. One trial detailed the method of blinding of assessors and were rated as low risk (17). Two studies rated as high risk had abscission data that was not used in their analyses, while they noted the drop-out rate of participant in the trials (14, 15). The remaining studies with all data included in the final analysis were classified as low risk. An overview of the results of the risk of bias analysis for the included studies was shown in Figure 2.

### Study characteristics

The characteristics of 17 RCTs included in the current meta-analysis were presented in Table 1. These studies were published between 2011 and 2021. All the participants in the study were older adults ≥ 60 years old, the vast majority of whom were recruited from communities or hospitals. The Baduanjin intervention in some studies were combined with other exercise, but the movement paradigm of Baduanjin in the studies included was basically consistent.

### Berg Balance Scale

**FIGURE 3:**
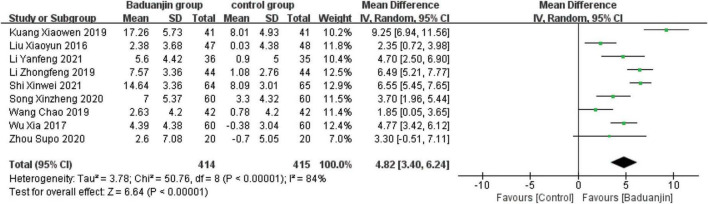
Forest plot of the meta-analysis of BBS. CI, confidence interval; SD, standard deviation.

Nine studies reported scores of BBS, with significant heterogeneity among these studies (χ^2^ = 50.76, df = 8; *P* < 0.00001, *I^2^* = 84%). Results from the sensitivity analysis showed that the exclusion of any single study did not influence the heterogeneity and mean difference (MD) among studies with the parameter of BBS. The analysis using a random effect model showed that BBS scores were significantly higher in Baduanjin than in control groups (MD = 4.82, 95% CI 3.40 to 6.24, *P* < 0.00001; Figure 3).

### Timed Up and Go

**FIGURE 4:**
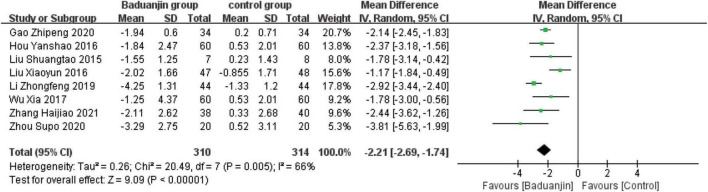
Forest plot of the meta-analysis of TUG. The green squares and horizontal lines indicate the study-specific mean difference and 95% CIs. The size of the green area reflects the study-specific statistical weight. The black diamonds represent the mean difference and 95% CIs of each subgroup and the overall population.

Eight studies reported TUG scores, with significant heterogeneity among these studies (χ^2^ = 20.49, df = 7, *P* = 0.005, *I*^2^ = 66%). Results from the sensitivity analysis showed that the exclusion of any single study did not influence the heterogeneity and MD among studies with the parameter of TUG. The analysis by a random effect model showed that TUG test scores were better in Baduanjin than in control groups (MD = −2.21, 95% CI −2.69 to −1.74, *P* < 0.00001; Figure 4).

### Eye Closed One Leg Standing Balance

**FIGURE 5:**
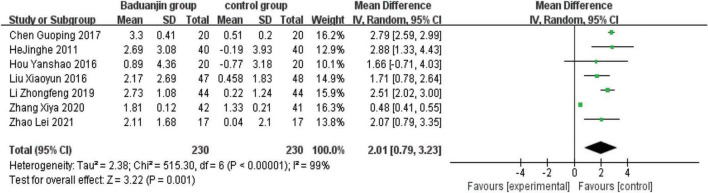
Forest plot of the meta-analysis of ECLSB. CI, confidence interval; SD, standard deviation.

Seven studies reported ECLSB scores, with significant heterogeneity among these studies (χ^2^ = 515.30, *P* < 0.00001, *I*^2^ = 99%). After the sensitivity analysis, it was speculated that the data of Zhang et al.’s study (15) was the main source of heterogeneity. Accordingly, the analysis was reperformed after the data of this study was excluded. The analysis by a random effect model showed that ECLSB test scores were better in Baduanjin than in control groups (MD = 2.01, 95% CI 0.79 to 3.23, *P* < 0.00001; Figure 5).

### Subgroup analysis

#### Health status of the participants

**FIGURE 6:**
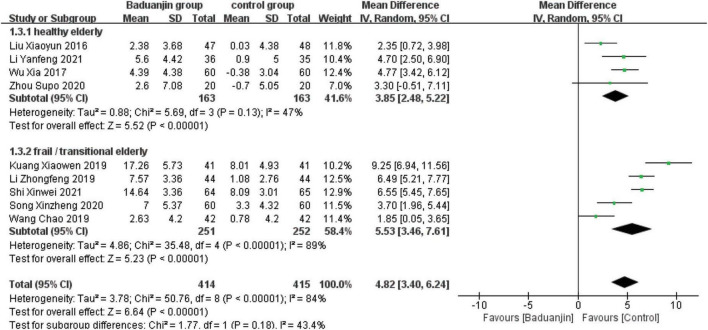
Forest plot of the subgroup analysis of BBS for the different health status. CI, confidence interval; SD, standard deviation.

**FIGURE 7:**
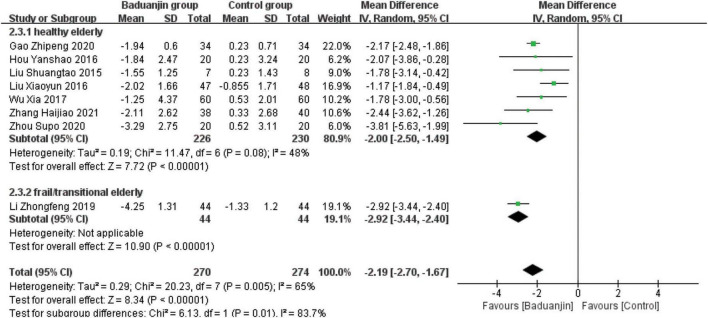
Forest plot of the subgroup analysis of TUG for the different health status. CI, confidence interval; SD, standard deviation.

**FIGURE 8:**
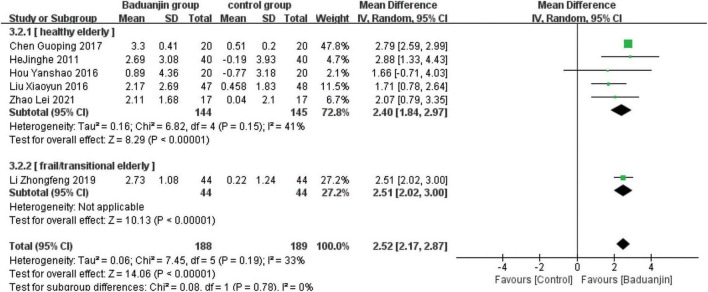
Forest plot of the subgroup analysis of ECLSB for the different health status. CI, confidence interval; SD, standard deviation.

**FIGURE 9:**
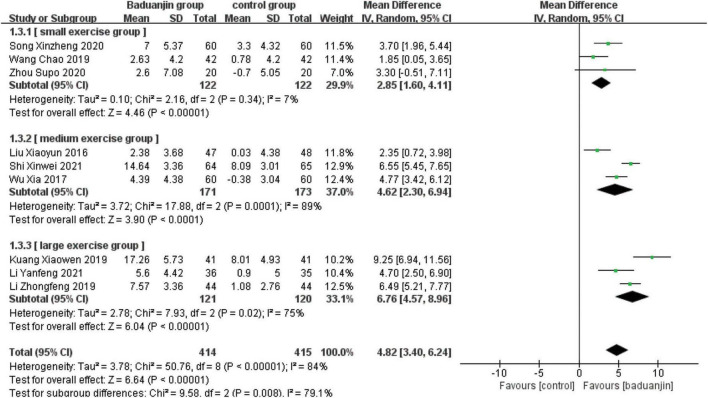
Forest plot of the subgroup analysis of BBS for the total exercise amount. CI, confidence interval; SD, standard deviation.

**FIGURE 10:**
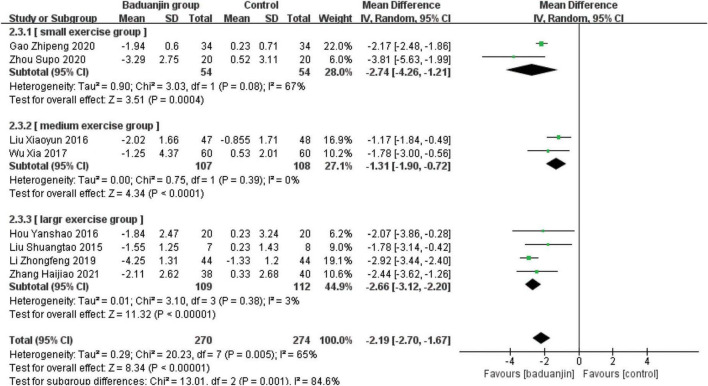
Forest plot of the subgroup analysis of TUG for the total exercise amount. CI, confidence interval; SD, standard deviation.

Due to the large heterogeneity among the included studies, as well as the differences in health status among study subjects, the RCTs that included BBS, TUG, and ECLSB scores were analyzed separately according to the health status of study subjects. The studies of healthy older subjects showed moderate heterogeneity (BBS: *I*^2^ = 47%; TUG: *I*^2^ = 49%; ECLSB: *I*^2^ = 41%). The analyses showed that BBS scores were better in Baduanjin groups than in control groups for both healthy (MD = 3.85, 95% CI 2.48 to 5.22, *P* < 0.00001) and frail/transitional (MD = 5.53, 95% CI 3.46 to 7.61, *P* < 0.00001) older adults. And the time reduction on the standing and walking test after the intervention was better in Baduanjin groups than in control groups of both healthy (MD = −2.00, 95% CI −2.50 to −1.49, *P* < 0.00001) and frail/transitional (MD = −2.92, 95% CI −3.44 to −2.40, *P* < 0.00001) older subjects. In addition, ECLSB scores were also significantly better in Baduanjin groups than in control groups of both healthy (MD = 2.40, 95% CI 1.84 to 2.97, *P* < 0.00001) and frail/transitional (MD = 1.48, 95% CI −0.51 to 3.47, *P* < 0.00001) older persons. High degree of heterogeneity was shown in the studies including the frail/transitional older subjects. All the above results were presented in Figures 6–10.

#### Total exercise amount

**FIGURE 11:**
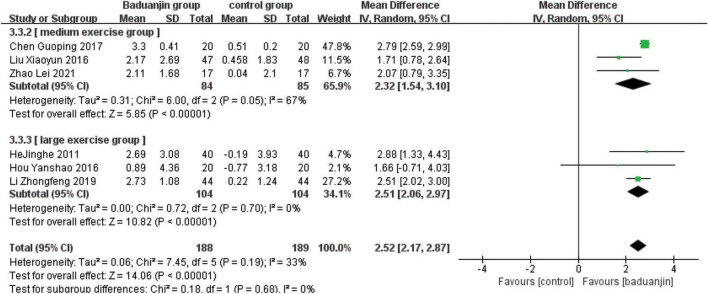
Forest plot of the subgroup analysis of ECLSB for the total exercise amount. CI, confidence interval; SD, standard deviation.

The results of subgroup analysis in different total exercise amounts are shown in Figures 9–11. The Baduanjin group had significant improvement than control group in BBS [SE: MD = 2.85, 95% CI (1.60, 4.11), *P* < 0.000 01; ME: MD = 4.62, 95% CI (2.30, 6.94), *P* < 0.000 1; LE: MD = 6.76, 95% CI (4.57, 8.96), *P* < 0.000 01], TUG [SE: MD = −2.74, 95% CI (−4.26, −1.21), *P* = 0.000 4; ME: MD = −1.31, 95% CI (−1.90, −0.72), *P* < 0.000 1; LE: MD = −2.66, 95% CI (−3.12, −2.20), *P* < 0.000 01] and ECLSB [ME: MD = 2.32, 95% CI (1.54, 3.10), *P* < 0.000 1; LE: MD = 2.51, 95% CI (2.06, −2.97), *P* < 0.000 01]. For the parameter of BBS, the subgroup pool effect size increased with the increase of total exercise amount, and there was the significant difference between SE and LE as shown in Figure 9.

#### Publication bias

**FIGURE 12:**
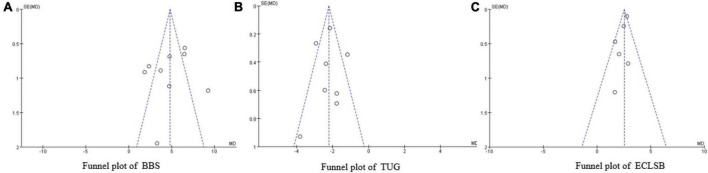
Funnel plots of BBS **(A)**, TUG **(B)** and ECLSB **(C)**. SE, standard error; MD, mean difference.

Funnel plots showed that the distribution of research points on the BBS, TUG, and ECLSB indices were generally symmetrical (Figures 12A–C), suggesting that there was little likelihood of publication bias.

## Discussion

Aging is associated with a decline of balancing ability caused by reductions in cognitive and motor function, increasing the likelihood of falls and associated injuries (28). Falls are one of the major cause affecting the QOL in the elderly. Muscle strength and balance are crucial elements of overall health, which help older adults lower their risk of falling. Practicing Baduanjin can increase flexibility and stability, as well as improving muscle strength and proprioception of the lower limbs (29).

As the outcome indicator for this systematic analysis, the measurement of BBS, TUG, and ECLSB is not complicated to conduct. BBS can systematically and quantitatively evaluate the balancing ability through a series of tests. TUG assesses the balancing ability of subjects in their daily activities by measuring the time required to sit up and walk. ECLSB measures vestibular function, hip flexion and extension muscle strength, with the subject’s vision blocked. BBS and TUG were shown to have good reliability and validity in previous research (30, 31). Comparatively, the evaluation standard for ECLSB is easily affected by external factors (32). Systematic evaluation of these three indicators in the present meta-analysis demonstrates that Baduanjin can effectively impact the balancing ability of older adults.

Physical flexibility and balance are strongly correlated, and increasing physical flexibility can enhance body coordination and balance, both of which are important factors in preventing falls (33). With regards to Baduanjin movements, it involves flexion and extension of the knee and ankles joints, internal and external rotation of the arms while keeping them flexed. Baduanjin exercise has been found to significantly enhance body flexibility in previous research (34).

In addition, some studies have examined how Baduanjin training affects the muscular strength in the lower limbs. Baduanjin’s persistent half-squat position will present constant challenges to the body’s balance and postural stability from the standpoint of its own movements. In particular, the feet must be level with the ground and cannot straddle the shoulder-width limit (similar to the oval area) (35). Some studies have demonstrated that Baduanjin exercise can significantly enhance spatial gait parameters such as stride length, walking speed, and stride frequency, which are also closely related to lower limb muscle strength and balance (36). After Baduanjin exercise, the vastus medialis muscle was found elevated, whereas the vastus lateralis muscle did not change significantly, by measuring the root mean square, integrated and average electromyogram (37). It’s possible that the 16-week intervention period in the research is not long enough.

As for the impact by the factor of training duration, this meta-analysis also revealed that the improvement of balancing ability is positively related to total exercise amount. Following the subgroup analysis based on total exercise amount, the heterogeneity with the subgroup is reduced, and it is basically shown that the larger the exercise amount, the stronger the effect of intervention. Such result was consistent with another study on another popular Chinese traditional exercise—Tai Chi (38). In this meta-analysis, it was shown that the improvement in the Baduanjin group’s BBS test followed the total exercise amount increased. But such a trend was not occurred in TUG and ECLSB. This outcome could be caused by the two factors. Firstly, the number of studies included in the analysis is relatively small, and the participant characteristics and intervention techniques used in each research varied from one another. In some studies, Baduanjin intervention was combined with other exercises such as balance or resistance exercise, while in some research older participants with specific medical issues, like stroke patients, were recruited. Secondly, when calculating the amount of exercise, it is defaulted that Baduanjin’s intensity is universal. The included research, however, may cover inconsistent approaches to execute Baduanjin’s intervention, such as instruction technique, movement modification, supervision process, etc., so the intensity could be altered. Although Baduanjin has a unified motion pattern when compared to various school of Taichi, the standard and quality of the movement completion could undoubtedly have an impact on the intensity of Baduanjin.

Baduanjin with low-to-moderate intensity is comparatively easy to learn and practice, making it ideal for improving balance in older persons. Besides the studies included based on the selecting criteria, the effect of Baduanjin on balancing ability of the elderly with varied health conditions has also been proved by numerous studies. In chronic stroke patients, Baduanjin is beneficial at enhancing balance, leg muscle strength, and flexibility. Additionally, Baduanjin can reduce the risk and frequency of falls in Parkinson’s disease patients by strengthening the lower extremities and enhancing balance (39).

Subgroup analysis according to the health status showed that Baduanjin was advantageous for all the older participants. The indicator of BBS and TUG differed significantly between healthy and frail/transitional older subjects. It was found that the heterogeneity was obviously lower for the subgroup of healthy older subjects than the whole, indicating that one of heterogeneity source could be related to the variations in the participants’ health status. In this study, the frail symptoms or diseases varied and only a small number of research examined frail or transitional older people. Thus, it was difficult to further evaluate and analyze each parameter because the pooled data on this subgroup had considerable heterogeneity and bias.

### Limitation

Some limitations should be acknowledged. Firstly, there may be omissions of research because only the databases of PubMed, Web of Science, The Cochrane Library, CNKI, Wan Fang, and SinoMed were searched owing to the limited conditions. Of the 17 selected studies, only one was from a study published outside China. Thus, the findings may not be generalizable to other nations and ethnicities. Secondly, the papers of poor quality were included, which had the lack of description of the blind and random distribution methods, the absence of analysis on the gender variable, and/or the failure to indicate whether subjects were missing or not in some trails. Thirdly, because of large heterogeneity across the included studies, only the random-effect model could be used, which had an impact on the findings.

## Conclusion

The current meta-analysis demonstrates that Baduanjin can enhance older adults’ ability to balance and that the effect of the intervention may vary depending on the participants’ health condition. In addition to further exploring the influencing factors of gender, age, and health status, more investigations are needed to determine the effects of Baduanjin on older adults from different cultures and ethnic groups.

## Data availability statement

The raw data supporting the conclusions of this article will be made available by the authors, without undue reservation.

## Author contributions

LG conceptualized and designed the study, coordinated and supervised data collection, and critically reviewed and revised the manuscript. ZL and WY collected data and wrote and revised the articles. All authors contributed to the article and approved the submitted version.
