# Supplementary material for: The effect of Baduanjin on the balancing ability of older adults: A systematic review and meta-analysis
Source: Front Med (Lausanne). 2022 Oct 28;9:995577. doi: 10.3389/fmed.2022.995577 (PMC9650403; doi:10.3389/fmed.2022.995577)
Supplement: Supplementary file 1 [file Data_Sheet_1.pdf]

Supplementary file

Search strategy:

1.1 For Web of Science

(TS=(Ba Duan Jin) OR TS=(eight section brocades) OR TS=(Ba-Duan-Jin) OR TS=(baduanjin)) AND (TS=(balance*) OR TS=(musculoskeletal equilibrium) OR TS=(fall) OR TS=(accidental falls)) AND TS=(elderly OR old*)

1.2 For PubMed

((ba duan jin) OR (eight section brocades) OR (Ba-Duan-Jin) OR (baduanjin)) AND((balance*) OR (musculoskeletal equilibrium) OR (fall) OR (accidental falls)) AND ((elderly) OR (old*))

1.3 For The Cochrane Library

ID Search

#1 (ba duan jin) OR (baduanjin) OR (ba-duan-jin) OR (eight section brocades) (Word variations have been searched)

#2 (balance*) OR (musculoskeletal equilibrium) OR (fall*) OR (accidental falls) (Word variations have been searched)

#3 (elderly) OR (old*) (Word variations have been searched)

#4 #1 AND #2 AND #3

1.4 For CNKI

SU=（’八段锦’）AND SU=（’老年人’+’老人’） AND FT=(‘平衡能力’+’跌倒’+’摔倒’)

1.5 For WanFang Data

主题: (("八段锦") and ("平衡能力" or "跌倒" or "摔倒") and ("老年人" or "老人"))

1.6 For SinoMed

"八段锦"[常用字段:智能] AND("平衡"[常用字段:智能] OR "跌倒"[常用字段:智能] OR "摔倒"[常用字段:智能]) AND("老年人"[常用字段:智能] AND "老人"[常用字段:智能])
